# A Solid-Contact Ion Selective Electrode for Copper(II) Using a Succinimide Derivative as Ionophore

**DOI:** 10.3390/s130404367

**Published:** 2013-04-02

**Authors:** Mihaela Dana Tutulea-Anastasiu, Deivy Wilson, Manel del Valle, Cristina Mihaela Schreiner, Igor Cretescu

**Affiliations:** 1 Faculty of Chemical Engineering and Environmental Protection, “Gheorghe Asachi” Technical University, 73 D. Mangeron Street, Iasi 700050, Romania; E-Mail: dtutulea@tuiasi.ro; 2 Sensors and Biosensors Group, Department of Chemistry, Universitat Autònoma de Barcelona, EdificiCn, Bellaterra 08193, Catalonia, Spain; E-Mails: dwilson25@gmail.com (D.W.); Manel.delValle@uab.es (M.V.); 3 Faculty of Electrical Engineering, “Gheorghe Asachi” Technical University, 53 D. Mangeron Street, Iasi 700050, Romania; E-Mail: cschrein@ee.tuiasi.ro

**Keywords:** copper, solid-contact electrode, PVC membrane, potentiometry, selectivity

## Abstract

All-solid-state sensors with polyvinyl chloride (PVC)-based membranes using off-the-shelf N-hydroxysuccinimide (NHS) and succinimide (Succ) ionophores were prepared using DOP (dioctyl phthalate) and NPOE (*ortho*-nitrophenyloctyl ether) as plasticizers. Good responses were obtained when NHS was used. The potentiometric response of the proposed electrode is independent of pH over the range 2–6. The electrode shows a fast response time of 0.25 s. The electrode exhibits a Super-Nernstian response, with 37.5 mV/decade, with a potentiometric detection limit of 4.4 μM. The proposed sensor revealed good selectivity towards a group of transition metal ions.

## 1. Introduction

Copper is known to be an essential element for health, but even in low concentrations, copper ions are toxic to all organisms and its determination is an important analytical task [1,2]. The increased accumulation of copper(II) in the environment from numerous industrial sources, poses a danger to public health [3]. Thus, the determination of trace amounts of copper(II) has become more and more important because of the increased interest in environmental pollution [4].

In order to estimate its deficiency or accumulation in various samples, sensitive, reproducible and accurate analytical methods are required [5]. Several methods including spectrophotometry [6,7] dispersive liquid–liquid microextraction [8,9], adsorptive stripping voltammetry [10], sequential injection analysis [11], high performance liquid chromatography [12], anodic stripping voltammetry [13,14] have been applied for the determination of copper(II) ions. Various copper(II) electrodes were developed using chalcogenide glass matrix [15–17]. Pyrrole [18], polyindole [19], salens [20], 3,6,9,14-tetrathiabicyclo [9.2.1]tetradeca-11,13-diene [21], aza-thioether crowns [22], 4-phenyl- 11-decanoyl-1,7-dithia-11-azacyclotetradecane-4-sulfide [23], 4-phenyl-4-sulfide-11-(1-oxodecyl)-1, 7-dithia-11-aza-4-phosphacyclotetradecane [24], copper sulphide [25], 5,6,7,8,9,10-hexahydro-2*H*-1,13,4,7,10-benzodioxatriazacyclopentadecine-11(4*H*,12*H*)-dione [26] were used as electroactive materials in different copper(II) selective membrane sensors.

Solid-contact ion-selective electrodes can provide very low detection limits. Moreover, due to the fact that these electrodes do not require an optimization of the inner filling solution, the method presents new advantages such as good mechanical stability and simplicity [27– 29], so different designs and/or disposable use are possible. Due to the need of selective and accurate determination of trace amounts of Cu(II) ions in water samples, many coordination compounds with high selectivity to metal ions have been used as ionophores, in the construction of copper-selective electrodes [30].

The metal-ligand interactions provide in consequence recognition mechanisms which can be used in the development of potentiometric sensors. It is well known that the nitrogen and oxygen donor atoms coordinate the transition metal ions to form metal complexes [31]. The ligands used in this study are involved for the first time in ion-selective electrodes development. The aim of this work was the preparation and testing of graphite-based epoxy electrodes [32] for the potentiometric determination of copper(II) ion.

## 2. Experimental Section

### 2.1. Materials and Measurements

All the chemicals were analytical grade or higher quality. The solutions were prepared using doubly distilled water. The components for membrane preparations (bis(2-ethylhexyl) phthalate (DOP), *o*-nitrophenyloctyl ether (NPOE), potassium tetrakis(4-chlorophenyl) borate (KpClPB), sodium tetraphenylborate (NaTPB), tetrahydrofuran (THF) and high-molecular-weight polyvinyl chloride (PVC)) and were obtained from commercial sources (Fluka, Buchs, Switzerland), being used as received. For pH control, sodium hydroxide (0.1 M) and nitric acid (0.1 M) were used. The solutions for the potentiometric measurements were prepared using the nitrate salts of the given cations (Merck, Darmstadt, Germany) and bidistilled water. The materials used to prepare the solid electrical contact were the epoxy resin components: Araldite M, Araldite M hardener, Araldite M accelerator (all from Fluka), and graphite powder (BDH, London, UK) as conductive filler.

### 2.2. Equipment

The emf measurements were performed with by a self-made data acquisition system consisting of 32 input channels equiped with differential instrumentation amplifiers (INA116, Burr-Brown, Tucson, AZ, USA) which adapted the impedance for each sensor. The emf measurements were performed against a double junction Ag/AgCl reference electrode (Thermo Orion 90-02-00, Waltham, MA, USA). Each channel was noise-shielded with its signal guard. The output of each amplified channel was filtered with a second order low pass active filter centered at a 2 Hz frequency and connected to an Advantech PC-Lab 813 A/D conversion card installed in a PC. The readings were acquired by using custom software developed by our group in Microsoft QuickBasic Version 4.5. For the pH adjustment, a Crison 2002 pH-meter (Crison, Barcelona, Spain) with a combination pH electrode (Ingold model 10/402/3092, Crison, Barcelona, Spain) was used.

### 2.3. Electrode Preparation

The potentiometric sensors used were all-solid-state ion selective electrodes (ISEs) with a solid electrical contact made from a conductive composite [32]. The epoxy mixture, used as supporting conductor, was obtained by mixing Araldite M, the hardener and the accelerator in mass ratios of 1:1:0.05. This resulting paste was mixed with graphite powder 1:1 mass ratio and then was introduced in the electrode body [33]. After curing, in the electrode body was made a cavity of 0.3 mm depth. The ion selective membrane solution was obtained combining plasticizers (63.3%), ionophores (1.9%), KTpClPB (approx. 0.5%) and PVC (30.7%) in THF (3 mL). Drops of this cocktail were deposited on the electrodes surface with a micropipette and let dry for 24 h. After this time, THF was evaporated and transparent membranes were obtained. Prior to first use, the prepared electrodes were conditioned in a 0.1 M Cu(II) solution for 24 h.

### 2.4. Characterization of the Sensors

ISEs were characterized for both considered cations by separate calibration procedures. They consisted in the recording of ISE potentials after cumulative microadditions of considered ions. The sensitivity corresponded to the slope of the linear response against the ion activity's logarithm. The ion activity coefficients in solution were calculated according to the Debye-Hückel formalism [34]. The ISEs lower detection limit (DL) was taken at the point of intersection of the two asymptotic behaviours of the calibration curve, as recommended by IUPAC [35].

## 3. Results and Discussion

The main analytical parameters of the electrodes including the detection limit, the linear response range, the pH effect, the response time and the selectivity to other ions were evaluated.

The potentiometric response of all solid state contact Cu(II)-selective electrodes prepared with DOP and NPOE was evaluated in the concentration range of 10^−8^ to 10^−2^ M, against a double distilled water blank.

**Figure 1.**
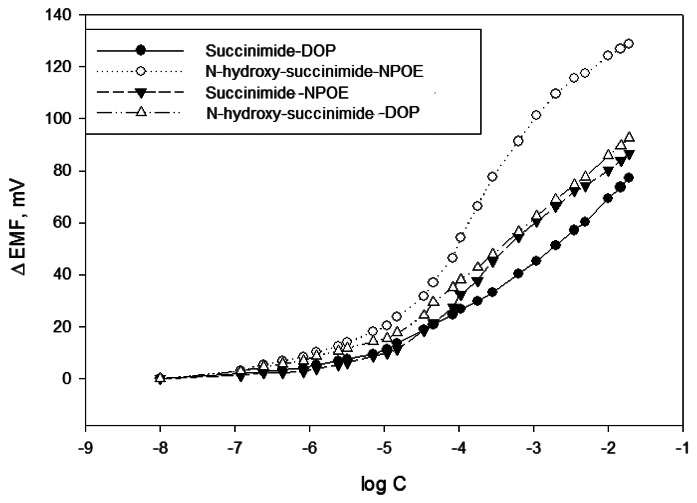
Potential response toward Cu(II) ions of the electrodes with the four potentiometric membranes evaluated.

ΔEmfs were plotted against log of activities of the Cu(II) ions, as shown in Figure 1. The sensitivity values were considered to be the slopes of the linear portion of the calibration graph, at a correlation coefficient R^2^ > 0.99.

When the membrane based on succinimide–DOP was used, the potential remained linear in the concentration range 10^−4^–10^−1^ M and a slope of 20.9/decade was observed. The summary of results for the different membranes tested is displayed in Table 1.

The combination of N-hydroxysuccinimide with NPOE plasticizer in particular shows a super-Nernstian slope of 37.5 mV/decade. This super-Nernstian slope could be explained by poor permeability and incomplete permselectivity of the membrane matrix for the copper ions. Another explanation can be that the different slope arises from the different stoichiometry of complexation reaction between Cu(II) and the ligand, or in the mixed equilibrium between chloride and ligand complexation of Cu(II). The first explanation seems more probable than the second one, since the same behavior is expected for both NHS and S ligands. In any case, the higher value of slope does not hinders in any way the determination of copper(II) ions in aqueous media.

The potential response of the proposed electrodes shows a linear response to the Cu(II) concentration in the range of 10^−5^ to 10^−2^ M. These characteristics together with the low detection limit make our formulation interesting among other recently reported Cu(II) ionophore-based sensors (see Table 1). Another advantage of the ligands involved in this study is that they are readily available commercially and rather cheap in comparison with other ligands utilized in previously published papers.

### 3.1. Effect of pH

**Figure 2.**
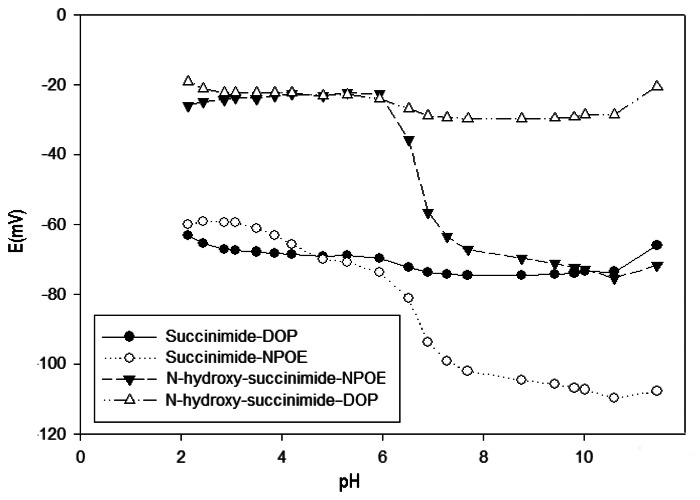
Effect of the pH on the response of the electrodes at 1 × 10^−3^ M Cu(II) concentration.

**Figure 3.**
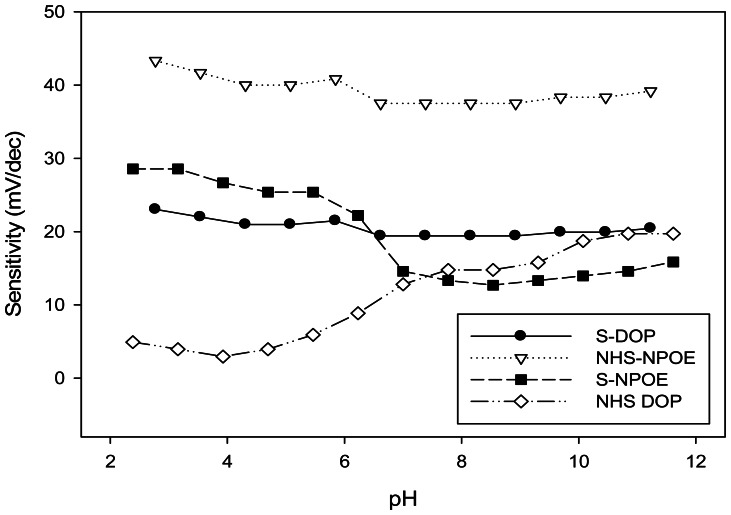
Effect of pH on the observed sensitivities for the four membranes evaluated.

The influence of pH on the potential response of the proposed sensors was studied over the pH range 2–12 (adjusted with HNO_3_ or NaOH) at two Cu(II) ion concentration values, 1.0 × 10^−3^ and 1.0 × 10^−4^ mol·L^−1^. Obvious changes of the potential values with the pH value are noticed, as presented in Figure 2 for a concentration value of 10^−3^ mol·L^−1^. A similar behavior, but with less effect by the pH change, was observed for the lower concentration. Essentially, the potentials remain unchanged in a pH range from 2 to 6 (Figure 3), which is considered to be the working pH range of the developed Cu(II) selective electrode. An increase of the potential could be explained due to the formation of hydroxyl complexes of Cu(II) in solution, while, at the lowest pH, certain protonation of the ligand can be ascertained. This usable pH range can be highlighted as one of the widest available among different ionophore-based Cu(II) sensors (Table 1).

The sensitivity values were calculated by substracting the emf values corresponding to the investigated values of the Cu(II) concentrations, 10^−3^ and 10^−4^ M, at different, well established pH values. The sensitivity plot against pH for the investigated ligands is plotted in Figure 3.

### 3.2. Response Time

**Figure 4.**
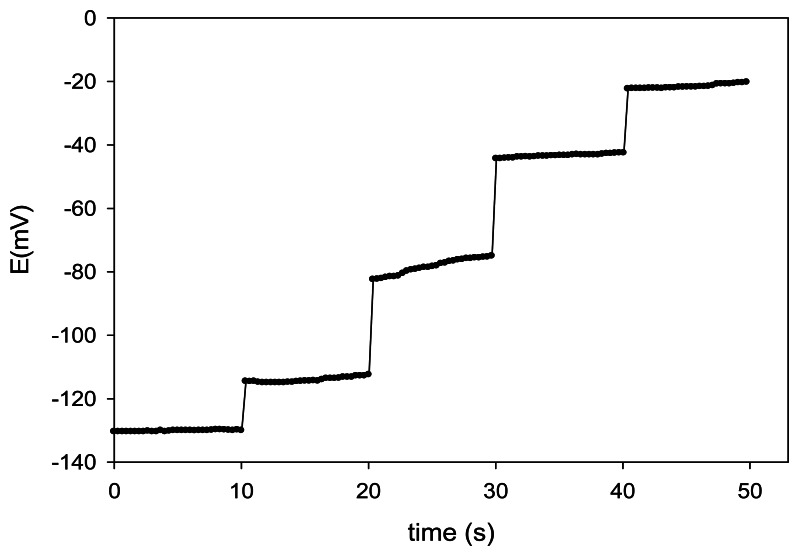
Dynamic response of N-hydroxysuccinimide–NPOE membrane electrode, with one decade step changes of concentration (10^−6^ to 10^−2^ M).

The response time is an important factor for an ion-selective electrode [39]. The dynamic response time was evaluated while changing the concentration of Cu(II) in solution over a concentration range from 10^−6^ to 10^−2^ mol·L^−1^, by measuring the potentials with the automatic data acquisition system (Figure 4).

In all cases, the response was very fast, not showing any time lag, which can be considered as an exponential charging time transient. For this reason, the response time of the developed ISEs was estimated to be *ca.* 0.25 s, additionally pointing out the rapid complex formation. Again, the prepared sensors show one of the fastest response rates among comparable ionophore-based Cu(II) sensors (Table 1).

### 3.3. Selectivity

The selectivity coefficient of the proposed electrodes was evaluated by using the fixed interference method (FIM) and it was determined from the potential measurements of solutions containing a fixed constant activity of the interfering ion (10^−4^ mol·L^−1^) and varying activity of primary ion [40].

The selectivity coefficient is calculated from the following equation:

(1) $$K_{i,j}^{\text{pot}}=a_{i}^{*}/{\left(a_{j}\right)}^{z_{i}/z_{j}}$$

where *a_i_, a_j_* = the activities of primary ion and respective of interfering ion; *z*_i_, *z*_j_ = their respective charges.

In the procedure followed a fixed concentration of interfering ions (1.0 × 10^−4^ mol·L^−1^) and a varying concentration of Cu(II) ion was used. The results of the interference study are summarized on Table 2, where the data from other research on Cu(II) ion selective electrodes were included, for the sake of comparison. *k_Cu,X_*

The electrodes with NPOE-plasticized copper(II) ion-selective membrane electrode showed better selectivity coefficients for different cations than that with DOP-plasticized membrane electrode. This suggests that the coordination of copper(II) ion by the ionophores can be related to the plasticizer type. It is also generally accepted, that NPOE plasticizer obtains improved performance for bivalent cation membranes. Especially, the N-hydroxysuccinimide-NPOE displayed better selectivity, improving with one order of magnitude or more the calculated selectivity coefficients.

From the selectivity coefficients it can be observed that the electrode membrane presents better selectivity to copper(II) ion than to the other cations. This demonstrates that the electrode membrane coordinates stronger the copper(II) ions.

It can be observed that the ligand containing nitrogen and oxygen atoms as coordination centers generated quite selective ionophores in NPOE-plasticized PVC membranes for copper(II) ion.

The stability of the developed sensors was tested by measuring the electrode potential as a function of time, by checking both the detection limits and the slopes of the calibration curve. A series of calibrations were carried out over a few weeks in Cu(II) solution. The long-term stability of the copper(II) electrode was excellent, with no significant change in the working range and slope observed after three months of use. After two weeks, the electrodes responses were at a level of 98% and after one month at around 96% of the initial values.

The repeatability of the ion selective electrode was investigated by measuring the response of a specific electrode several times under the same set of conditions. The copper(II) ion selective electrode was tested for eight consecutive days and displayed good repeatability in a range of ±0.3 mV. The RSD value for N-hydroxysuccinimide–NPOE membrane was 1.64%. The slope of calibration curve obtained for this electrode was found to decrease slightly after several uses, which may be attributed to surface contamination.

## 4. Conclusions

Solid-contact potentiometric Cu(II) selective sensors were developed by incorporating off-the-shelf N-hydroxysuccinimide and succinimide ionophores in a PVC matrix, using DOP (dioctyl phthalate) and NPOE (*ortho*-nitrophenyloctyl ether) as plasticizers. The best performances were obtained for the N-hydroxysuccinimide version. The sensors work in a wide pH range 2–6 with a response time of less than 1 second. The main advantages of the developed electrodes can be summarized as: very fast response time, low cost, long life time and wide working pH range. Beside these characteristics, the electrodes are very easy to prepare, show low detection limits, good reproducibility, repeatability, and selectivity for Cu(II) over some metal ions, which recommend its use for a wide range of analytical applications.

## Figures and Tables

**Table 1.** Performance characteristics of Cu(II) selective sensors, formulated in this work and from the literature.

**Membrane**	**Sensitivity (mV/dec)**	**Detection Limit (M)**	**Working Concentration Range (M)**	**pH Working Range**	**Response Time (s)**	**Lifetime (Months)**
N-hydroxy-succinimide-DOP	24.23	4.8 × 10^−6^	10^−4^ to 10^−2^	2–6	1 s	3
N-hydroxy-succinimide-NPOE	37.46	4.4 × 10^−6^	10^−4^ to 10^−2^	2–6	0.25 s	3
Succinimide-DOP	20.90	9.6 × 10^−6^	10^−4^ to 10^−2^	2–6	1 s	3
Succinimide-NPOE	25.35	7.3 × 10^−6^	10^−4^ to 10^−2^	2–6	1 s	3
Reference [36]	29.2	3.6 × 10^−6^	10^−5^ to 10^−2^	3.5–6.5	18 s	-
Reference [37]	30.0	3.0 × 10^−6^	10^−5^ to 10^−1^	1–3	8 s	4
Reference [38]	29.6	2.4 × 10^−6^	10^−5^ to 10^−1^	3–5	30 s	3

**Table 2.** Selectivity coefficients of PVC membranes evaluated in this work.

**Membrane**	**log*k****_Cu,X_*				

	**Ca^2+^**	**Co^2+^**	**Ni^2+^**	**Zn^2+^**	**Pb^2+^**
N-hydroxysuccinimide-DOP	−1.06	−1.26	−1.19	−0.73	−0.81
N-hydroxysuccinimide-NPOE	−2.41	−3.55	−1.49	−2.06	−0.59
Succinimide-NPOE	−1.78	−0.89	−0.91	−0.59	−0.070
Succinimide-DOP	−0.25	−1.039	−1.030	−0.32	−0.071
Reference [36]	−1.21	−1.09	−1.12	−1.09	-
Reference [37]	−1.09	-	-	−0.15	−0.79
Reference [38]	0.032	0.044	0.35	0.019	0.04
